# Non-coding RNA regulation of integrins and their potential as therapeutic targets in cancer

**DOI:** 10.1007/s13402-022-00752-y

**Published:** 2022-12-13

**Authors:** Tristan Joseph Verhoeff, Adele F. Holloway, Joanne L. Dickinson

**Affiliations:** 1 Menzies Institute for Medical Research, College of Health and Medicine, University of Tasmania, Hobart (Tasmania), Australia; 2 Tasmanian School of Medicine, College of Health and Medicine, University of Tasmania, Hobart (Tasmania), Australia

**Keywords:** lncRNA, Long non-coding RNA, Epigenetics, ncRNA, Integrins

## Abstract

**Background:**

Integrins are integral to cell signalling and management of the extracellular matrix, and exquisite regulation of their expression is essential for a variety of cell signalling pathways, whilst disordered regulation is a key driver of tumour progression and metastasis. Most recently non-coding RNAs in the form of micro-RNA (miRNA) and long non-coding RNA (lncRNA) have emerged as a key mechanism by which tissue dependent gene expression is controlled. Whilst historically these molecules have been poorly understood, advances in ‘omic’ technologies and a greater understanding of non-coding regions of the genome have revealed that non-coding RNAs make up a large proportion of the transcriptome.

**Conclusions and Perspectives:**

This review examines the regulation of integrin genes by ncRNAs, provides and overview of their mechanism of action and highlights how exploitation of these discoveries is informing the development of novel chemotherapeutic agents in the treatment of cancer. MiRNA molecules have been the most extensively characterised and negatively regulate most integrin genes, classically regulating genes through binding to recognition sequences in the mRNA 3′-untranslated regions of gene transcripts. LncRNA mechanisms of action are now being elucidated and appear to be more varied and complex, and may counter miRNA molecules, directly engage integrin mRNA transcripts, and guide or block both transcription factors and epigenetic machinery at integrin promoters or at other points in integrin regulation. Integrins as therapeutic targets are of enormous interest given their roles as oncogenes in a variety of tumours, and emerging therapeutics mimicking ncRNA mechanisms of action are already being trialled.

## Introduction

Cell surface molecules involved in adhesion are vital to cancer progression as they mediate cell interactions with their surrounds and bridge these interactions to intracellular signals that change gene expression. A particularly important group of these molecules are the integrins. Human integrins comprise 24 protein heterodimers, composed from 18 α and 8 β subunits [1], with extracellular, transmembrane, and cytoplasmic-signalling domains. Each integrin recognises and binds specific extracellular matrix components with different affinities, allowing the cell to respond to its environment. Integrins influence multiple biological processes relevant to metastasis including, adhesion, proliferation, differentiation, migration, and angiogenesis. Namely, integrin binding to the extracellular matrix (ECM) recruits signalling and cytoskeletal proteins at adhesion complexes, mediating different signalling and cytoskeletal responses with activation occurring by both extrinsic (outside-in) or intrinsic (inside-out) pathways [2].

Several integrins have been shown to be important in cancer progression including the formation of distant metastases associated with poor survival. There has been extensive characterization of the integrin family of adhesion molecules in tumour progression, reporting a myriad of expression changes during tumour progression (reviewed [3, 4]). However we are only just beginning to unravel the exquisite mechanisms regulating gene expression, and how they are perturbed in tumour development. Examples include the integrin αvβ3 which is strongly expressed in normal breast epithelia and highly expressed in breast cancer (BC) bone metastases [5]. Integrin ITGA2 (of α2β1) is initially a tumour suppressor gene in BC and prostate cancer (PC), its loss promoting metastasis [6–8], however metastatic cells increase ITGA2 expression to facilitate metastasis to bone [8–11]. Both α2β1 and αvβ3 adhesion also guide cofilin activation via focal adhesion kinase (FAK) [12, 13], directly bind matrix metalloproteinases (MMPs) for invasion and angiogenesis [14–17], and form a vascular endothelial growth factor 2 (VEGFR2) − integrin complex that enhances pro-angiogenic/integrin signalling [17–22]. Integrins α3β1 and α5β1 are also involved in BC metastasis [9], while α5β1 in lung cancer, through is binding to fibronectin, was shown to be essential in lung cancer proliferation, adhesion, and metastasis [23].

Regulatory mechanisms controlling integrin expression are diverse and complex and a large body of evidence now exists showing that epigenetic mechanisms play an important role in regulation of integrins. Epigenetic mechanisms encompass heritable changes in DNA expression that do not change the genetic sequence. DNA is organised around nucleosomes with nucleosome positioning and chemical modifications contributing to transcriptionally accessible euchromatin, transcriptionally inaccessible repressive and compact heterochromatin, and boundary regions [24]. By adjusting how genes are accessed, epigenetic modifications act as the ‘command centre’ providing instructions to the DNA, guiding not just how regions are transcribed, but also how they interact with other regulatory regions of the DNA such as enhancers and repressors. As different epigenetic modifications interact with each other they also act in a multi-dimensional manner, with some modifications acted upon by others, so that they establish higher-order patterns of gene expression. As this occurs in a cell-type specific manner, epigenetic mechanisms are dominant regulators of the cell specific transcriptome. Epigenetic mechanisms can be broadly grouped into DNA methylation, histone modifications, nucleosome (histone) remodelling, and non-coding RNAs (ncRNAs) [25, 26]. Multiple integrin genes are known to be regulated in cancer by DNA methylation at their promoter regions including *ITGA1*, *ITGA2*, *ITGA4*, *ITGA7*, and *ITGA9* [27–31], and some (e.g., *ITGA2*) are known to be regulated by histone modifications including H3K27me3 [12, 32]. More recently it is becoming clear that non-coding RNAs play an important role in the regulation of integrins and there is growing realisation that they are surprisingly diverse and potentially mediate the exquisite regulation of integrins, perhaps even more so than DNA methylation and histone modifications.

The ncRNAs are diverse regulators of epigenetics, transcription, and translation, that provide another facet of gene regulation. NcRNAs, and lncRNAs (long non coding RNA) in particular, account for most of the genes in human cells (at least 60−70% of transcribed genes being lncRNAs, versus approximately 22% protein coding genes) [33]. MicroRNA (miRNA), lncRNAs (including circular RNA), and piwi interacting RNA (piRNA) are all associated with cancer progression, acting as both tumour suppressors or oncogenes [34–38]. A burgeoning area of interest is ncRNAs (miRNA and lncRNA) and their regulation of integrin genes in a cancer context, and the diverse mechanisms by which they regulate integrin gene expression.

### MiRNA biogenesis and function

MiRNAs are small RNAs (averaging 22 bp) and are transcribed as a primary transcript (pri-miRNA), and often with multiple miRNAs transcribed together in the pri-miRNA as a miRNA family [39]. Canonically, pri-mRNA is cleaved by the Drosha/DGCR8 complex into one or several pre-miRNA molecules. These pre-miRNAs are then exported from the nucleus and processed by Dicer-1 into 5p and 3p mature miRNAs [39]. MiRNAs regulate mRNA molecules by complementary sequence interactions most commonly through the 3′−UTR (untranslated region) of the mRNA, regions termed miRNA response elements (MREs). The 3p or 5p miRNAs combine with Argonaute proteins (AGO1–4) in RNA induced silencing complex (RISC), the miRNA is loaded into AGO as a duplex, the loaded strand (5p or 3p) complemented by its passenger strand which is later removed before binding to the mRNA [39].

Multiple miRNAs can engage single mRNAs and conversely, a given miRNA can engage 100s of mRNAs, allowing great complexity in regulatory abilities, with miRNAs acting as tumour suppressor genes (TSGs) or oncogenes depending on their target [25]. MiRNAs have received significant attention in recent years with many regulating integrins by acting as TSGs and opposing pro-oncogenic integrins. The known list of validated miRNA–mRNA interactions is actively growing with several added each year.

### Direct miRNA–integrin mRNA interactions

**Fig. 1:**
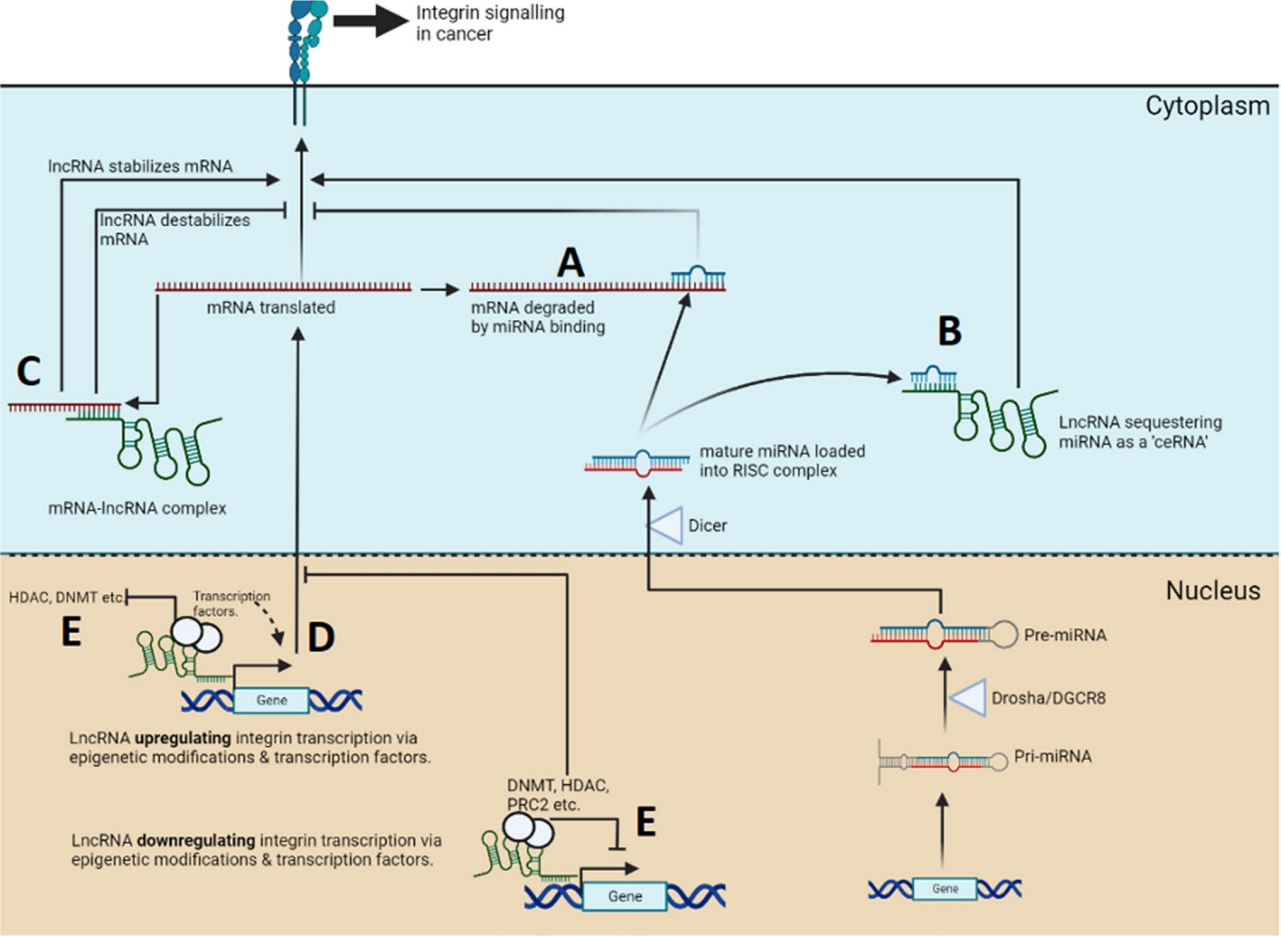
Schematic showing possible mechanisms by which ncRNA (in this case lncRNA and miRNA) regulate integrin genes. **A**–**E** relate to mechanisms discussed earlier in this review. Created with BioRender.com

Amongst the plethora of miRNAs now described, there are several that are notable master regulators in key pathways. MiR-31-5p is a master regulator of integrins in metastatic BC and PC cells, impairing metastatic adhesion and spreading on ECM components collagen, laminin, and vitronectin [40]. MiR-31-5p directly represses mRNAs of integrins *ITGΑ2*, *ITGA5*, *ITGAV*, and *ITGB3* through 3′-UTR interactions, and while *ITGB1* and *ITGB5* are not targeted directly, the loss of their heterodimer partners results in a reduction in their protein expression [40]. Many integrin genes are targeted by multiple miRNAs, *ITGA2*, *ITGB1*, and *ITGB3* appear to have the most distinct miRNAs targeting their mRNAs (see Table 1), but this may be biased by the large body of literature examining these key integrins. Using *ITGA2* as an example, most miRNAs targeting ITGA2 mRNA have TSG functions and are reduced in expression within cancers to permit *ITGA2* driven adhesion, proliferation, anti-apoptosis, invasion, migration, and epithelial-to-mesenchymal transition (EMT) (per Table 1). In contrast, miRNAs may be oncogenic in circumstances where the loss of an integrin enhances metastasis (where migration is not dependent on integrin adhesion). For example, in BC soft tissue (lymph node) metastases and the luminal MCF-7 cell line, the over-expression of miR-373-3p in BC reduced *ITGA2* expression, and the reduced expression of the integrin resulted in increased migration but not invasion (as invasion is necessarily integrin dependent) [41]. For some miRNA interactions with integrins (e.g., miR-135b-5p and ITGA2) the miRNA needs to be associated with two sites in the mRNA 3′UTR simultaneously to fully downregulate the integrin [42]. The mechanisms by which miRNA downregulate integrin mRNA transcripts is represented in Fig. 1A.

**Table 1:** MiRNAs regulating integrin genes in cancer with validated interactions

Integrin	MiRNA	Cancer	Impact of miRNA on cancer	Ref
*ITGA1*	miR-1298-5p	Glioma	TSG, anti-proliferative/pro-apoptotic	[43]
*ITGΑ2*	miR-373-3p	BC	TSG, anti-metastatic/adhesive	[41, 44]
	miR-107	PaC	TSG, anti-metastatic/invasive	[45]
	miR-30a-5p	GC	TSG, anti-proliferative/migratory	[46]
	miR-31-5p	BC/PC	TSG, anti-invasive/metastatic	[40]
	miR-16-5p	PTC/CRC	TSG, anti-proliferative/invasive/metastatic	[47, 48]
	miR-135b-5p	GC	TSG, anti-proliferative/pro-apoptotic	[42]
	miR-128	Ost	TSG, anti-invasive/metastatic/EMT	[49]
	miR-103a-3p	PTC	TSG? Not validated	[50]
	miR-206	BC	TSG, anti-metastatic/invasive/proliferative	[51]
	miR-138-5p	GC	TSG, anti-proliferation/metastasis/EMT	[52]
	miR-195-5p	nscLC	TSG, anti-proliferation/migration/invasion	[53]
	miR-34c-3p	nscLC	TSG, anti-metastatic/invasive	[54]
*ITGA3*	miR-223-3p	PC	TSG, anti-invasive/migratory/proliferative	[55]
	miR-33b-5p	CRC	TSG, anti-proliferative/migration	[56]
*ITGA5*	miR-31-5p	BC/PC	TSG, anti-invasive/metastatic	[40]
	miR-92a-3p	OvC	TSG, anti-invasive/migratory/proliferative	[57]
	miR-128-3p	Glioma	TSG, anti-proliferative/invasive/migration	[58]
	miR-30c-5p	CRC	TSG, anti-proliferative	[56]
*ITGA6*	miR-143-3p	CeC	TSG, anti-proliferative/invasive/migratory	[59]
	miR-498-5p	HCC	TSG, anti-proliferative/invasive/migratory	[60]
	miR-488	Ost	TSG, anti-metastatic	[61]
*ITGA9*	miR-152-3p	CM	TSG, anti-metastatic	[62]
	miR-324-5p	RMS	TSG, anti-proliferative	[63]
	miR-7	RMS	TSG, anti-proliferative/invasive/migratory	[63]
*ITGA11*	miR-516b-5p	nscLC	TSG, anti-proliferative/migratory/invasive	[64]
*ITGAV*	miR-31-5p	BC/PC	TSG, anti-invasive/metastatic	[40]
*ITGB1*	miR-124-3p	OSCC	TSG, anti-adhesive/migratory	[65]
	miR-183-5p	HeLa cells	TSG, anti-invasive/migratory	[66]
	miR-223-3p	PC	TSG, anti-angiogenic	[55, 67]
	miR-29a/b/c-3p	HNSCC/PaC	TSG, anti-metastatic/invasive	[68, 69]
	miR-493-5p	nscLC	TSG, anti-proliferative/migratory/invasive/pro-apoptotic	[70]
	miR-1226-3p	HCC	TSG, anti-metastatic	[71]
	miR-34c-3p	nscLC	TSG, anti-metastatic/invasive	[54]
*ITGB3*	miR-let-7a	Melanoma	TSG, anti-invasive	[72]
	miR-let-7c-5p	CRC	TSG, anti-proliferative/metastatic	[56]
	miR-let-7d-5p	CRC	TSG, anti-proliferative/metastatic	[56]
	miR-30c-5p	CRC	TSG, anti-proliferative/metastatic	[56]
	miR-31-5p	BC/PC	TSG, anti-invasive/metastatic	[40]
	miR-590-3p	NPC	TSG, anti-metastatic	[73]
	miR-1275	NPC	TSG, anti-metastatic	[73]
	miR-338-3p	LC	TSG, anti-proliferative/metastatic/invasive	[74]
*ITGB4*	miR-532-5p	LAd	possibly TSG, not yet validated	[75]
*ITGB5*	miR-221-5p	HCC	TSG, anti-proliferative/metastatic/invasive	[76]

Validated interactions are those where the specific 3'-UTR region has been investigated for functionality. Referenced studies often do not state − 5p/-3p identity of miRNAs, with this confirmed on miRbase (http://www.mirbase.org/ ) where possible. Referenced studies have employed human cell lines, analysed tumour samples, with an additional in vivo test in a mouse model included in some instances. Cancer subtype abbreviations: BC = breast cancer, CeC = cervical cancer, CM = cutaneous melanoma, CRC = colorectal cancer, GC = gastric cancer, HCC = hepatocellular carcinoma, LAd = lung adenocarcinoma, LC = lung cancer, nscLC = non-small cell lung cancer, NPC = nasopharyngeal carcinoma, Ost = osteosarcoma, OvC = ovarian cancer, PaC = pancreatic cancer, PTC = papillary thyroid carcinoma, RMS = rhabdomyosarcoma, SCC (OSCC) = squamous cell carcinoma (Oral SCC)

### DNA mutations as mediators of miRNA–integrin mRNA interactions

The interaction between a miRNA and a MRE is dependent on sequence complementarity, and therefore sequence changes (genetic lesions or polymorphisms) to a response element may decrease how strongly an interaction occurs, possibly permitting unchecked upregulation of an otherwise downregulated gene. Such mutations have been reported, though infrequently, in integrin genes. Three beta group integrins have such sequence changes. The *ITGB3* rs3809865 T/A variant was significantly associated with oral squamous cell carcinoma risk and reduced integrin expression, the SNP being in a putative MRE for miRNAs miR-26b, miR-330, and miR-324-5p [77]. *ITGB4* rs743554 G/A allele, at a miR-34a MRE, was associated with BC progression, reduced survival, and metastasis [78]. The *ITGB5* A/C variant of rs2675, which occurs at a putative MRE for mir-192, miR-215, miR-449, and miR-504, had increased risk of bladder cancer, but no relationship with recurrence-free survival [79]. As for alpha group integrins, *ITGAV* with a GC variant of rs11902171 had a decreased risk of PC, presumably by disrupting the interactions of predicted micro RNAs (miR-382, miR-30a-3p, and miR-30e-3p) [80]. The *ITGΑ2* 3′−UTR contains SNPs rs6898333 and rs6880055 that have been associated with PC risk, and these SNPs flank MREs including that of miR-373-3p [41, 81].

Mutations can also occur within the miRNA gene itself to influence integrin gene regulation. Mutation of the seed sequence of miR-184 inhibits its ability to compete with miR-205 for a MRE in the ITGB4 mRNA 3′-UTR, though this was not described in a cancer context [82].

### miRNA regulation of integrins via upstream targets

The interactions of miRNAs with integrin genes can also occur by a fascinating variety of other mechanisms. A relatively common, and likely important mechanism in cancer progression, are the several TSG miRNAs modulating integrin expression by targeting upstream activators and epigenetic machinery controlling integrin gene transcription. MiR-124-3p directly downregulates Talin 1 mRNA, blocking the activation of β1 integrins and thus their downstream (often oncogenic) signalling including kinase cascades, EMT progression, and pro-invasive MMP2/9 expression [83]. For context, Talin 1 is a central activator of β1 (ITGB1) integrins, with a p35 − Cdk5 − Talin1^− phosorylated^ − β1 pathway essential for PC metastases where integrin adhesion was essential (e.g., bone metastases) [84]. MiR-760 and miR-199a-5p also downregulated ITGB1 indirectly, the former downregulating MOV10 mRNA, an RNA-binding protein that stabilizes ITGB1 mRNA (the miRNA acting as an anti-proliferative/pro-apoptotic TSG in pancreatic cancer) [85]. The latter downregulates ETS1 mRNA, preventing the Ets-1 transcription factor upregulating pro-metastatic ITGB1 in BC [86]. In a mechanistically different manner, miR-101 directly downregulated *DNMT3B*, preventing *ITGΑ1* promoter methylation and thus upregulating α1β1 [87].

These diverse examples of how miRNAs regulate integrin dependent pathways, merely suggest at the immense number of possible miRNAs and pathways that may be relevant to integrins when not considering those miRNAs that directly target the integrin mRNAs themselves.

### Long noncoding RNA biogenesis and function

Long non-coding RNAs (LncRNAs) are a far more diverse and poorly understood group of transcripts when compared to miRNAs, with 20,000–68,000 potentially functional lncRNA genes in the human genome, far outnumbering miRNA or protein coding genes [33, 88]. Although they remain largely uncharacterised, they are emerging as significant players in tumour development. LncRNAs are ncRNAs greater than 200 bp in length, and often have relatively low levels of expression, but this is often tissue specific and these transcripts may have a wide variety of regulatory roles in the nucleus and cytoplasm (several of which are related to cancer progression as will be discussed) [34, 89]. Circular RNA (circRNA) are also more than 200 bp in length and are often classed with lncRNA, but are circular rather than linear and may operate via different mechanisms [34, 89]. Many lncRNAs directly engage proteins and nucleic acids to guide epigenetic machinery and transcription factors to gene promoters (or sequester them away from targets) in *cis* or *trans* [90–92]. The functional role of lncRNAs in a given cancer (as a TSG or an oncogene), may depend on the role of their target integrins in a particular tumour type as an oncogene/TSG and may indeed depend on the stage of cancer given the dynamic expression of many integrins throughout tumour development. Herein, integrin regulating lncRNAs (including circular RNAs) are categorised as follows: **2.1.1** lncRNAs acting as competitive endogenous RNAs (ceRNAs) via miRNA regulation, **2.1.2** lncRNAs that directly complement a mRNA transcript, **2.1.3** lncRNAs guiding transcription factors to integrin promoters, and **2.1.4** lncRNAs mediating integrins via interactions with chromatin or recruitment of epigenetic machinery. Each category is defined below, with examples listed in Table 2.

**Table 2:** LncRNAs regulating integrin genes

Integrin gene	lncRNA (miRNA target if ceRNA)	Mechanism (+ or −)	TSG/oncogene (cancer)	Ref
ITGA1	HOTTIP	TFR (+)	Cartilage regulation	[87]
	Hsa_circ_0110757 (miR-1298-5p)	ceRNA (+)	Onc (Glioma)	[43]
ITGA2	I2ALR	ACo (−)	TSG (BC)	[30]
	PICSAR	Possible CI (−)	Onc.ProMet (SCC)	[93]
	LINC00355	TFR (+)	Onc (CRC)	[94]
	LOC284454	CI (−)	TSG (BC)	[95]
	MALAT1	CI/TFR (+)	Onc.ProMet (BC)	[96]
	UBE2CP3 (miR-138-5p)	ceRNA (+)	Onc.ProMet (GC)	[52]
	SLC25A25-AS1 (miR-195-5p)	ceRNA (+)	Onc (nscLC)	[53]
	UCA1 (miR-107)	ceRNA (+)	Onc (PaC)	[45]
ITGA2b	MALAT1	CI/TFR/SpSc (+)	Onc.ProMet (BC)	
ITGA3	MALAT1	CI/TFR (+)	Onc.ProMet (BC)	[96]
	ITGB8-AS1 (miR-33b-5p)	ceRNA (+)	Onc.ProMet (CRC)	[56]
ITGA5	PICSAR	Possible CI (−)	Onc.ProMet (SCC)	[93]
	NEAT1 (miR-128-3p)	ceRNA (+)	Onc (Glioma)	[58]
	ITGB8-AS1(likely miR-30c-5p)	ceRNA (+)	Onc (CRC)	[56]
	ABHD11-AS1	ceRNA?* (+)	Onc.ProMet (CRC)	[97]
ITGA6	OIP5-AS1 (miR-143-3p)	ceRNA (+)	Onc (CeC)	[59]
	PRR34-AS1 (miR-498-5p)	ceRNA (+)	Onc (HCC)	[60]
	SNHG16 (miR-488)	ceRNA (+)	Onc.ProMet (Ost)	[61]
	BORG	TFR (+)	Onc.ProMet (BC)	[98]
ITGA9	HOXA11-AS (miR-152-3p)	ceRNA (+)	Onc.ProMet (CM)	[62]
ITGA11	FEZF1‑AS1 (miR‑516b‑5p)	ceRNA (+)	Onc (nscLC)	[64]
ITGAV	AY927503	CI (+)	Onc.ProMet (HCC)	[92]
	MALAT1	CI/TFR (+)	Onc.ProMet (BC)	[96]
	KCNQ1OT1 (miR-26a-5p)	ceRNA (+)	NA	[99]
ITGB1	BLNCR	Unknown	NA (epidermal differentiation)	[100]
	PICSAR	CI? (−)	Onc. ProMet (SCC)	[93]
	SOCS2-AS1	Unknown* (+)	Onc (Glioma)	[101]
	TUG1 (miR-29c-3p)	ceRNA** (+)	Onc.ProMet (PaC)	[69]
	NR2F1-AS1 (miR-493-5p)	ceRNA (+)	Onc (nscLC)	[70]
	ZFPM2-AS1 (miR-1226-3p)	ceRNA (+)	Onc.ProMet (HCC)	[71]
	LINC01354	Unknown (+)	Onc.ProMet (Ost)	[102]
ITGB2	ITGB2-AS1	ACo (+)	Onc (BC)	[103]
ITGB3	MALAT1	CI/TFR (+)	Onc.ProMet (BC)	[96]
	FAM225A (miR-590-3p; miR-1275)	ceRNA (+)	Onc.ProMet (NPC)	[73]
	ITGB8-AS1 (miR-30c-5p; let-7c-5p; let-7d-5p)	ceRNA (+)	Onc.ProMet (CRC)	[56]
ITGB4	AL139385.1 (miR-532-5p)	ceRNA (+)	Onc (LAd)	[75]
ITGB5	RNF185-AS1 (miR-221-5p)	ceRNA	Onc.ProMet (HCC)	[76]

LncRNA mechanism: + indicates up regulation and - indicates down regulation of integrin. The name/symbol for the lncRNA used here is that used in the referenced study for clarity rather than the standardized names on LncBase or GeneCards. LncRNA are mechanistically grouped into: ACo = antisense complex, ceRNA = competitive endogenous RNA, CI = chromatin interaction, SpSc = splicing scaffold, TFR = transcription factor recruitment. *=lncRNA cytoplasmic and regarded as likely a ceRNA, but a miRNA targeting both wasn’t identified. **=likely ceRNA identified and confirmed to engage the lncRNA, but the integrin 3′-UTR site was not tested. Cancer sub-type abbreviations: BC = breast cancer, CeC = cervical cancer, CM = cutaneous melanoma, CRC = colorectal cancer, GC = gastric cancer, HCC = hepatocellular carcinoma, LAd = lung adenocarcinoma, LC = lung cancer, nscLC = non-small cell lung cancer, NPC = nasopharyngeal carcinoma, Ost = osteosarcoma, OvC = ovarian cancer, PaC = pancreatic cancer, PTC = papillary thyroid carcinoma, RMS = rhabdomyosarcoma, SCC (OSCC) = squamous cell carcinoma (Oral SCC). Other abbreviations: NA = not applicable, Onc = oncogene, ProMet = pro-metastatic

#### LncRNAs as competitive endogenous RNAs

Perhaps the most commonly reported mechanism of lncRNA gene regulation is by acting as competitive endogenous RNAs (ceRNAs), sequestering miRNAs that would otherwise bind integrin mRNA 3′−UTR regions (and thus upregulating the integrin). This mechanism is depicted in Fig. 1B and occurs via the lncRNA binding to a matching MRE on the target mRNA [104]. This process is complex with miRNAs having multiple targets with different affinities, and ceRNAs in turn possibly having multiple miRNA sites with varying affinities. The strength of competing miRNA site affinities may determine how much ceRNA is required to compete for different response elements on mRNAs, with this also depending on the relative expression of the miRNA, ceRNA, and mRNA [104]. However, it must be emphasized that ceRNAs are contentious and the extent of their functionality is debated. The role of many putative ceRNAs has been questioned as given the widespread distribution of any individual MRE across a large number of transcripts, it appears difficult to conceive how variations in level of expression of a lncRNA (which are already low expressed in general) will change the biological effect of a miRNA. The experimental evidence for and against the ceRNA hypothesis is reviewed elsewhere [105].

In recent years the number of putative ceRNA interactions involving integrins has increased greatly (tabulated in Table 2), and those miRNAs involved have already been discussed earlier. Notably, possible ceRNAs are often cytoplasmic, as are the miRNAs they sequester [106], consistent with localization reported for most putative ceRNAs listed in Table 2. However ceRNAs are not always cytoplasmic, with some nuclear localized lncRNAs acting as ceRNAs, for example LINC00336 binding miR-6852 in lung cancer [107]. Typically these putative ceRNA-acting lncRNA molecules are oncogenic, permitting oncogenic integrin signalling by removing miRNAs that would otherwise downregulate the integrin: *ABHD11-AS1* permits ITGA2 − p-AKT − p-P85(PI3K) − p-AKT1 signalling for proliferation, invasion, and migration [97]; *HOXA11-AS* permits ITGA9 induced cutaneous melanoma progression and EMT [62]; *NEAT1* facilitates ITGA5 induced p-FAK signalling, cyclin D1 (proliferation) and antiapoptotic prevention of cleaved caspase 3 formation [58]; *SNHG16* increases ITGA6 mediated EMT [61]; and *TUG1* facilitates ITGB1 mediated EMT and MMP2/9 upregulation [69].

Several putative ceRNAs are reported to sequester multiple miRNAs. The lncRNA *UCΑ1* is reported to sequester at least five miRNAs (miR-107, miR-126, miR-182, miR-204, and miR-206) that can be either oncogenic miRNAs (*UCA1* thus acting as a TSG) or TSG miRNAs (*UCA1* thus an oncogene) [108–111], with the lncRNA upregulating integrin *ITGA2* by sequestering one of these miRNAs (miR-107 in PC) [45].

Regarding other integrin genes, *ITGB8-AS1* was found to upregulate *ITGA3*, *ITGA5*, and *ITGB3* by sequestering four different miRNAs (see Table 2), however while *ITGA5* was regulated by the lncRNA, its miRNA was not confirmed (luciferase and rescue assays failed to confirm the ITGA5 interacting miRNA miR-30c-5p as a target of the ceRNA) [56].

#### LncRNA-mRNA interactions

LncRNAs often engage mRNAs directly via complementary interactions between the sequences, the RNA–RNA duplex coordinating with RNA binding heterogeneous nuclear ribonucleoproteins (hnRNPs) to form lncRNA − hnRNP − mRNA complexes. These complexes either stabilize mRNA and promote translation of oncogenes or destabilize mRNAs (downregulating the target gene) [112–114]. This mechanism is illustrated on Fig. 1C.

Interestingly, the binding sites are often in the 5′−UTR and 3′−UTR, e.g., lncRNA *RP11* downregulated SIAH1 and FBXO45 mRNAs via interactions with the CDS and 3′−UTR regions of the mRNAs [114], while all 5 predicted interaction sites for *I2ALR* were in the ITGA2 mRNA UTR [30]. This indicates that regulatory functions of these regions may compete with miRNAs. However exactly why the mRNA is stabilized or degraded remains unclear, and difficult to predict *in silico*.

Examples of this mechanism of integrin regulation are limited. *ITGB2-AS1*, transcribed in antisense to *ITGB2*, upregulated the integrin via a 231 bp complementary region to the mRNA, this upregulation resulted in BC progression [103]. Integrin ITGA2 mRNA was recently shown to be downregulated by lncRNA *I2ALR*, transcribed in antisense from a promoter adjacent to the integrin gene, which acted as a TSG against the oncogenic *ITGA2*. The mechanism of action was hypothesized to be mRNA to lncRNA complementary interactions (in 5′ and 3′−UTR regions) in coordination with some RNA binding proteins [30].

#### LncRNAs guiding transcription factors to gene promoters

A limited number of lncRNAs have been demonstrated to upregulate integrins by recruiting traditional DNA binding transcription factors to gene promoter elements, thus acting more as transcriptional regulators rather than through epigenetic mechanisms.

LncRNA *LINC00355* interacted with the *ITGA2* promoter and recruited transcription factor GTF2B, upregulating *ITGA2* to promote proliferation and metastasis of colon cancer [94]. The oncogenic lncRNA *MALAT1* upregulated several integrins to promote metastasis in BC, and while this was likely through unspecified chromatin interactions at promoters, the lncRNA was generally co-localized with transcription factors Sox5 and Sox9 among others (discussed later) [96]. As an overall mechanism, this is depicted in Fig. 1D.

In the normal physiological context of chondrogenic differentiation, lncRNA *HOTTIP* blocks expression of its bidirectional transcript HoxA13, preventing the HoxA13 transcription factor upregulating *ITGA1* expression [87]. The downregulation of *HOTTIP* also coordinates with the upregulation of miR-101, which downregulates *DNMT3B*, preventing methylation of *ITGA1*, and thus enabling transcription and access by transcription factors such as HoxA13 [87]. This latter example demonstrates a different means of lncRNA transcription factor control of an integrin, blocking the expression of a transcription factor rather than guiding its localization (this may be considered a mechanism distinct from the other).

#### LncRNA-chromatin interactions and recruitment of epigenetic machinery

Many lncRNAs have been implicated in gene regulation by interacting with the chromatin, including the modification of chromatin or DNA by recruiting or blocking epigenetic machinery. For example, lncRNAs *EcCEBP* and *Dali* block DNMT1 to upregulate genes in *cis* or *trans* respectively, the latter binding transcription factors at active genes whereupon the lncRNA blocks DNMT1 [90, 115, 116]. By contrast lncRNAs *Dum* and *Dacor1* bind DNMTs to guide their methylation [117, 118], and *lncRNA-p21* guides DNMT1 as well as suppressive histone methyl transferases [90, 119]. Other lncRNAs (such as *FOXP1-IT1*) upregulate targets in *cis* by inhibiting histone deacetylases and removing repressive histone linker variants to promote histone acetyl transferase (HAT) and RNA Polymerase II access [91, 92]. The ability of lncRNAs to guide Polycomb repressive complex 2 (PRC2) may be important in cancer. PRC2 coordinates with PRC1, and deposits H3K27me3 modifications to epigenetically silence regions of the genome [120]. Many lncRNAs also engage PRC2 and guide H3K27me3 mediated gene repression. Notable examples being competition between *XIST*/*TSIX* in X-chromosome inactivation or global PRC2 repositioning by lncRNA *HOTAIR* in cancer to upregulate oncogenes and downregulate TSGs [121, 122]. Other research suggests that lncRNAs engage PRC2 promiscuously, with RNA titrating PRC2 away from areas of high transcription and bringing it in proximity of chromatin in areas of low transcription [123].

Regarding integrins, their regulation through this grouping of lncRNAs is diverse, as may be expected. LncRNA *MALAT1* in BC promotes proliferation, metastasis, and adhesion by upregulating multiple integrin genes (*ITGΑ2, ITGA2b, ITGA3, ITGAV*, and *ITGB3*) and their associated focal adhesion pathways, as well as cytokine receptor genes [96]. *MALAT1* and lncRNA *NEAT1* coordinated their chromatin interactions to bind to active chromatin of the transcription start sites (TSS) and transcription termination sites (TTS) of genes, but also were enriched at nuclear speckles/paraspeckles where they formed splicing scaffolds for *ITGA2b* and other genes [96, 124]. LncRNA *LOC284454* in BC acted as an anti-proliferative/migratory TSG and downregulated *ITGΑ2* via direct engagement to chromatin as well as p68 [95]. The lncRNA *AY927503* upregulated integrin *ITGAV* (for hepatocellular carcinoma proliferation, survival, and metastasis) by engaging promoter histone linker H1.2, 1.3, and 1.4 and removing histone linker variant H1FX (via NCL chaperone), inducing transcriptionally active histone modifications (H3K9/14ac and H3K4me3), and decreasing suppressive histone modifications such as H3K27me3, and permitting RNA Polymerase II access [92]. Notably *AY927503* regulation of *ITGAV* was dependent on a small (150 bp) domain of the lncRNA engaging H1FX specifically [92]. Presumably some part of *AY927503* would be responsible for sequence recognition of the *ITGAV* promoter. Finally, lncRNA *BORG* directly bound the TRIM28 epigenetic mediator and guided it to the *ITGA6* promoter in BC stem cells (and metastatic MDA-MB-231 BC cells) to promote α6 upregulation dependent self-renewal, stemness, tumorigenicity, and metastasis [98]. TRIM28 can either down- or up-regulate genes, for the former it is recruited to promoters via binding to KRAB-ZNF (Krüppel-associated box domain zinc finger) transcription factors, with TRIM28 then complexing with epigenetic machinery (e.g., HDAC, HP1, SETDB1, and NuRD) that downregulate transcription [125]. In the latter TRIM28 complexes with CBF-A which engages promoter FTS-1 elements to enhance transcription, typically for EMT progression [125]. As a mechanism, the guiding or blocking of epigenetic machinery by lncRNAs at integrin genes is depicted in Fig. 1E.

Epigenetic machinery regulating lncRNAs can also upregulate integrins by controlling upstream regulators of integrins. For example the TSG lncRNA *HOXD-AS1* (downregulated to promote CRC progression) recruited PRC2 to the *HOXD3* promoter, silencing it with H3K27me3 histone modifications, thus preventing the HOXD3 transcription factor from upregulating the oncogenic *ITGB3* [126]. Given the number of steps in a regulatory pathway such as this, these pathways may be far more common yet difficult to identify and test.

### LncRNAs regulating integrins through uncertain mechanisms

Further lncRNAs regulate integrin genes by unknown mechanisms. In these instances, the mechanisms were not investigated by study authors, or they were unable to be investigated due to problems encountered while trying to test mechanisms. In squamous-cell carcinoma (SCC) lncRNA *PICSAR* downregulated ITGA2, ITGA5, and ITGB1 mRNAs and SRC kinase (*c-Src*), thus reducing α2β1 and α5β1 col I and fibronectin adhesion and increasing metastasis, and permitting increased adhesion independent migration [93]. This study did not propose or test an underlying mechanism, but the expression pattern would be consistent with the lncRNA directing a suppressor to the integrin gene (e.g., epigenetic machinery). lncRNA *LINC01354* in osteosarcoma upregulated ITGB1 mRNA (and increased invasion, metastasis, and EMT through this integrin) in a manner consistent with a ceRNA (i.e., lncRNA expression correlating with the integrin gene) but no investigation of the mechanism was made [102]. LncRNA *BLNCR* was transcribed in antisense from a shared promoter with *ITGB1*, with the lncRNA localized in the cytoplasm and both genes correlating in expression, consistent with a ceRNA mechanism [100]. However, the mechanism was not investigated (knockdown of ITGB1 induced a slight reduction in BLNCR but this was due to reduced integrin signalling and promoter activity) and KD of BLNCR could not be achieved by the study authors [100].

LncRNA PICART1, transcribed antisense to *ITGA3* (adjacent promoters) was not investigated as a direct regulator of ITGA3, but it downregulated all components of the p-AKT/p-GSK3B/β-catenin signalling cascade as a TSG, a pathway known to be regulated by ITGA3 [127]. PICART1 may regulate ITGA3 in a similar manner to how I2ALR regulates ITGA2.

### Integrin regulating ncRNAs as therapeutic targets

LncRNAs and miRNAs are not current targets of chemotherapy but their apparently widespread action across different cancers has resulted in strong interest in their possible use as chemotherapy targets or biomarkers of disease. However, the incomplete understanding of these transcripts (regarding both their functionality and basic characterization) presently hinders realisation of this potential. Antisense oligonucleotides (ASOs) are one such therapy, these are synthetic single stranded deoxyribonucleotides (DNA of 12–30 bps) that bind their target RNAs (complementary base pairing) and decrease expression via RNase H (which degrades the RNA in a DNA–RNA heteroduplex). ASOs can also sterically block translation or modify RNA-splicing, by inducing exon skipping or conversely inclusion, as is the case for the FDA approved eteplirsen and nusinersen which are approved for the treatment of specific forms of Duchenne muscular dystrophy and spinal muscular atrophy respectively (Reviewed—[128]). The so-called 2nd and 3rd generation ASOs use chemical modifications to improve stability and limit toxicity and aberrant immune responses. Many use 2′ modifications to the sugar (as RNA) but this limits RNase H activity and thus chimeric ASO ‘gapmers’ have a central region of DNA bases (to recruit RNase H) flanked by 2′ modified RNA bases. Eteplirsen and nusinersen harbour 2′ modifications, but are not ‘gapmers’ and don’t require RNase H activity (see review articles—[128, 129]).

The ASO gapmers, Inotersen and Volanesorsen are two of the few therapeutics approved that act via RNase H, binding to the transthyretin mRNA (knocking down a defective form of the transcript) and apolipoprotein CIII mRNA, in transthyretin amyloidosis and familial chylomicronemia syndrome respectively [130, 131]. Inotersen and Volanesorsen follow a typical design of 10 central DNA bases, flanked by 5 RNA bases with 2′-O-2-methoxyethyl modifications, with a phosphorothioated backbone [130, 131].

Therapeutics that act as small interfering RNAs (siRNA or RNAi) are also in use. These are introduced in the double-stranded form, comprise only RNA bases, and bind to and degrade mRNAs via recruiting a RISC complex, similar to a native miRNA [128]. Frequently these RNA-based therapeutics have chemical modifications to reduce immune responses [129]. The 21 bp long Patisiran is one of the few approved therapeutic siRNA molecules and is used to treat hereditary transthyretin amyloidosis by knocking down transthyretin mRNA (both mutant form and wild type) produced in the liver [132].

Whilst the large size of many lncRNAs precludes their suitability for use as therapeutics themselves, both ASOs (including ASO ‘gapmers’) and siRNA-based therapeutics may be employed to specifically target oncogenic lncRNA molecules. Various ASOs, siRNAs, and similar molecules have been extensively used in preclinical studies to explore ncRNA functionality and regulation [129], and the targeting of lncRNAs to trigger transcriptional changes has been demonstrated, e.g., targeting lncRNA *SMN-AS1* to prevent it silencing the neighbouring *SMN2* gene *via* PRC2 recruitment [133].

Efficient delivery of ASO/siRNAs to specific tissues and cancers is challenging as the molecules are large, prone to degradation, and are hydrophilic. Innovative approaches such as chemical modifications, and the use of lipid nanoparticles, oligonucleotide conjugates, and oligonucleotide coated metallic nanoparticles for targeted delivery, have been developed to overcome some of these limitations [129]. For example, the aforementioned siRNA Patisiran, is intravenously targeted specifically to the liver, the siRNAs is encapsulated in a lipid nanoparticle which associates with endogenous apolipoprotein E (ApoE) to facilitate uptake by liver hepatocyte ApoE receptors [132]. Similarly, ASOs designed to knockdown the androgen receptor (AR) mRNA in PC can be specifically delivered using intravenously injected iRGD (internalizing Arg-Gly-Asp peptide)-liposomes. These liposomes successfully target the tumours, and the liposomes’ chemical conjugates allowed tissue specific targeting to PC bone metastases, with AR expression successfully reduced in bone metastases relative to healthy bone [134]. PEGylated lipid-PLGA nanoparticles have also been shown to deliver ASO gapmers specifically to bone marrow-derived mesenchymal stem cells [135]. These approaches to tissue specific delivery of ASOs/siRNAs could be readily adjusted to target integrin regulating ncRNA pathways.

## Conclusion

Overall, ncRNAs regulate the majority of integrin genes in a wide range of cancer subtypes, and are likely to be critical for the functioning of these integrin genes in cancer progression. These molecules regulate the integrins in complex pathways, however knowledge of their mechanisms of action and delineation of their scope of activities will assist in rationalizing approaches to target these molecules therapeutically. The recent development of tissue specific delivery mechanisms for ASOs/siRNAs, highlights that the mechanisms to deliver ncRNA targeting therapies to specific tissues or the tumours themselves offers great promise. This is of particular importance given the dearth of effective therapies for the treatment of many tumour types once they have progressed to metastatic disease.

## Data Availability

Not applicable.
